# Size of the abductor hallucis muscle in older women with hallux valgus

**DOI:** 10.1186/1757-1146-7-S1-A57

**Published:** 2014-04-08

**Authors:** Karen J Mickle, Christopher J Nester

**Affiliations:** 1 Biomechanics Research Laboratory, University of Wollongong, NSW, 2522, Australia; 2 Centre for Health Sciences Research, University of Salford, Salford, M6 6PU, UK

## 

Toe deformities are highly prevalent in older people with up to 74% of older men and women having some degree of hallux valgus [1]. Despite the well documented hypotheses that atrophied, or weak toe flexor muscles are associated with the formation of toe deformities [2], there has been little evidence to support this theory. Only one study has directly compared the toe flexor strength of individuals with toe deformities to those without, revealing that older people with hallux valgus have reduced hallux strength compared to those without the deformity [3]. Therefore, to further investigate the pathomechanics of hallux valgus, this study aimed to determine whether the size of the abductor hallucis muscle differed in older women with and without hallux valgus deformity.

Forty-four older adults (60+ years) were recruited to participate in the study. Each participant had their feet assessed by the Chief Investigator (KJM), with hallux valgus severity rated using the Manchester Scale [4]. The abductor hallucis muscle was imaged using a GE Venue 40 US with a 6-9 MHz transducer [5]. Muscle cross-sectional area (CSA) was measured using Image J software with the assessor blinded to group allocation. Ten participants (all women) were classified as having moderate or severe hallux valgus and their muscle size was compared to 10 age and BMI matched women without any hallux deformity.

**Figure 1:**
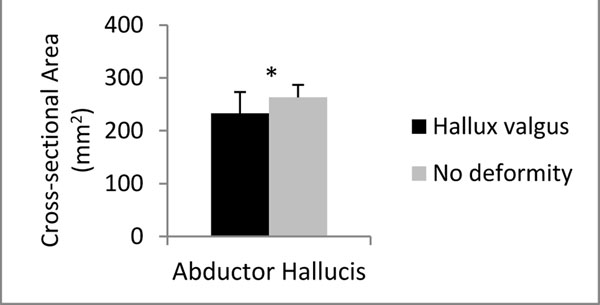
Mean (± SD) abductor hallucis CSA in women with and without hallux valgus deformity. * indicates significant difference.

The older women with moderate-severe hallux valgus were found to have a significantly reduced cross-sectional area of the abductor hallucis muscle (p < 0.05; Figure 1). This may suggest that muscle weakness and atrophy is associated with the development or progression of hallux valgus, however further longitudinal studies are required to confirm this notion. Further research is also required to determine whether strengthening the toe flexor muscles results in hypertrophic changes to muscle morphology and these results highlight the need to investigate whether strengthening the intrinsic toe muscles could reduce the incidence and severity of toe deformities.
